# Genotyping-by-sequencing of Canada’s apple biodiversity collection

**DOI:** 10.3389/fgene.2022.934712

**Published:** 2022-08-25

**Authors:** Zoë Migicovsky, Gavin M. Douglas, Sean Myles

**Affiliations:** ^1^ Plant, Food, and Environmental Sciences, Faculty of Agriculture, Dalhousie University, Truro, NS, Canada; ^2^ Genome Centre, McGill University, Montréal, QC, Canada

**Keywords:** Malus domestica, Malus sieversii, apple, germplasm collections, woody perennials, fruit collections, Genotyping-by-Sequencing

## Introduction

There are over 10,000 named apple (*Malus* X. *domestica* Borkh) cultivars (Way et al., 1991), but most apple production relies on a small number of elite cultivars. These elite cultivars are also the primary source of breeding material used when generating new cultivars (Migicovsky et al., 2021a). Apple production and improvement could greatly benefit from incorporating more diverse cultivars for purposes including disease resistance (Khan and Korban, 2022) and unusual fruit attributes (Migicovsky and Myles, 2017). Before leveraging such potential benefits, it would first be necessary to comprehensively assess phenomic and genomic diversity across diverse apples.

Apples, like many other woody perennials, are obligately outcrossing and highly heterozygous, meaning that in order to retain genetically identical individuals over time, clonal propagation is used (Miller and Gross, 2011). As a result, living germplasm collections are critical for the *ex situ* conservation of these woody perennials. However, these collections may also serve other purposes including as genetic mapping populations for crop improvement (Migicovsky et al., 2019).

Canada’s Apple Biodiversity Collection (ABC) is one of the most diverse collections of apples in the world, which was designed to enable genetic mapping. The ABC is located at the Agriculture and Agri-Food Canada (AAFC) Kentville Research and Development Centre in Nova Scotia, Canada. A comprehensive description of the ABC was recently published (Watts et al., 2021). Briefly, the collection consists primarily of apple accessions from the United States Department of Agriculture (USDA) Plant Genetic Resources Unit apple germplasm collection in Geneva, New York, USDA, but also includes additional accessions from Canada.

Although there are currently only 1,119 accessions (planted in duplicated) in the ABC, initial grafting efforts included additional accessions in order to ensure backups were available. As a result, the dataset presented here includes some accessions that are not planted in the ABC. However, because sequencing data were available for these accessions, we have retained them. The accessions primarily belong to the cultivated apple, *M. domestica*, but also include *Malus sieversii* (Ledeb.) M. Roem., the primary progenitor species of *M. domestica*.

In addition to phenotypic descriptions of the ABC, sequencing the accessions in the collection provides a valuable resource not only for researchers working on the collection, but for those studying apples more broadly. With this in mind, we report and make publicly available genotyping-by-sequencing (GBS) data for over 1,000 apple accessions from the ABC.

## Materials and methods

Young leaf tissue was collected from all accessions in the ABC and DNA was extracted using commercial kits. DNA was sequenced using GBS (Elshire et al., 2011) with ApeKI and PstI-EcoT22I restriction enzymes. GBS libraries were sequenced using Illumina Hi-Seq 2000 technology, using 100 bp single-end reads across 1,240 unique accessions. The mean read depth per accession was 2,159,274 for ApeKI and 2,287,219 for PstI-EcoT22I, while the median values were 1,917,843 for ApeKI and 2,166,834 for PstI-EcoT22I. By accession, read depth ranged from a minimum of 2,907 to a maximum of 17,985,988 for ApeKI and from 1,114 to 17,331,828 for PstI-EcoT22I. For both enzymes combined, the minimum read depth was 300,743 with a maximum of 35,317,816 (mean: 4,446,493, median: 4,039,234). The raw sequence data were deposited in the Short Read Archive under NCBI Bioproject ID PRJNA636391.

Single nucleotide polymorphisms (SNPs) were called using three different SNP calling pipelines: GATK (v3.7) (McKenna et al., 2010), SAMtools (v1.3) (Li et al., 2009), and TASSEL (V5.2.32) (Bradbury et al., 2007), using reference genome GDDH13 Version 1.1 (Daccord et al., 2017). A visual summary of the SNP calling and imputation pipeline is included in Supplementary Figure S1.

Failed raw reads were removed using Illumina’s CASAVA-1.8 FASTQ filter (http://cancan.cshl.edu/labmembers/gordon/fastq_illumina_filter/). Next, the GBSX toolkit (v1.3) (Herten et al., 2015) was used to deconvolute reads for the GATK/SAMtools pipelines. GATK/SAMtools pipeline reads were then trimmed using BBMAP (v35.82) (https://sourceforge.net/projects/bbmap/) to remove nucleotides with low quality (<20) from the 5′ end of each read, and to remove any trimmed reads that were <30 nucleotides. Reads with full enzyme cut sites were removed using BBMAP, as they were likely chimeric sequences. Reads were then pooled across runs and enzymes into one file per accession.

GATK/SAMtools pipeline reads were aligned to the reference genome separately using BWA (v0.7.12) (Li and Durbin, 2009). GATK’s HaplotypeCaller algorithm was run on each separate accession’s reads to generate genomic variant call format files (GVCFs), which were combined by random groups of 50 accessions using GATK’s “CombineGVCFs” program. SNPs were called from these combined GVCF files using GATK’s “GenotypeGVCFs” command. For the SAMtools pipeline reads, SAMtool’s “mpileup” command was run on reads for each accession, and then SNPs were called using the bcftools (v1.3) “call” command. For the TASSEL pipeline reads, SNPs were called using an alternative method of the TASSEL 5 GBS v2 Pipeline for each enzyme separately. Two SNP tables resulted, one for each enzyme used, and these were combined using a custom Perl script that preferably kept SNPs from the PstI-EcoT22I read set, as they had higher read coverage on average. The TASSEL SNPs were filtered to contain SNPs with a minimum minor allele frequency (MAF) of 0.01, and then the SNP sets from all three different SNP callers (GATK, SAMtools and TASSEL) were filtered using PLINK (v1.07) (Purcell et al., 2007; Purcell, 2009) to remove indels, and sites with more than two alleles. Following this filtering step, the GATK/SAMtools SNPs were also filtered for a MAF of 0.01 using PLINK.

SNPs were imputed for each caller separately using LinkImputeR (Money et al., 2017) at a maximum position/sample missingness of 70% and a minimum depth of four reads, resulting in imputation accuracies/correlation values of 0.9558/0.8761 (GATK), 0.9526/0.8696 (SAMtools), and 0.9556/0.8347 (TASSEL). Following imputation, SNP counts for each caller were 165,418 (GATK), 195,667 (SAMtools), and 226,821 (TASSEL). SNPs were pooled by merging the three VCF files and when SNPs overlapped across callers, one SNP was randomly chosen resulting in a final SNP set with 22.64% of SNPs from GATK, 30.23% from SAMtools, and 47.14% from TASSEL. The resulting SNP set consisted of 278,224 SNPs across 1,175 unique accessions.

Genotyping of an additional 8 markers was conducted using high resolution melting (HRM) on a LightScanner HR384 (BioFire). These markers included *NAC18.1*, *PG1*, *ACO1*, and *ACS1* as previously described (Migicovsky et al., 2021b). In addition to these four texture-related markers, the *Ma1* marker for acidity (Bai et al., 2012) was also genotyped, as well as three scab resistance markers, *Rvi2*, *Rvi6*, and *Rvi15* (Jänsch et al., 2015). Primers for all HRM markers are listed in Supplementary Table S1.

Since NAC18.1 was genotyped using both GBS and HRM, the GBS SNP was removed using PLINK and replaced with the HRM genotype calls. The markers which had been genotyped using HRM were merged using the --merge function in PLINK (v1.07) (Purcell et al., 2007; Purcell, 2009) into the SNP table at the appropriate position, as determined using NCBI BLAST (v2.2.31) using the apple reference genome GDDH13 Version 1.1, as described above (Daccord et al., 2017). Insertion/deletions were recoded as SNPs. Missing data were imputed using LinkImpute with k = 7, l = 12, and the resulting accuracy was 0.9501 (Money et al., 2015)**.** The final SNP set consisted of 278,231 SNPs across 1,175 unique accessions.

SNP density and distribution were examined including the number of SNPs per chromosome, inter-SNP distance, and the MAF distribution. The 278,231 SNP set was pruned for linkage disequilibrium (LD) using PLINK (v1.07) (Purcell et al., 2007; Purcell, 2009). To do this, we used a window size of 10 SNPs, removing pairs of SNPs with *R* ^2^ > 0.5, before shifting the window by three SNPs and repeating (PLINK command: -indep-pairwise 10 3 0.5). This filtering resulted in a pruned set of 180,075 SNPs that were used for principal components analysis (PCA) with TASSEL (Bradbury et al., 2007). Principal components (PCs) 1 and 2, which combined explained a total of 8.9% of the variance in the genomic data, were plotted. All data visualizations were performed using R version 4.1.0 (R Core Team, 2021) with the R package ggplot2 (v3.3.5) (Wickham, 2016). Metadata description of the 1,175 apple accessions genotyped for this study, as well as genomic PCs 1 to 10, are included in Supplementary Table S2.

## Conclusion

**FIGURE 1:**
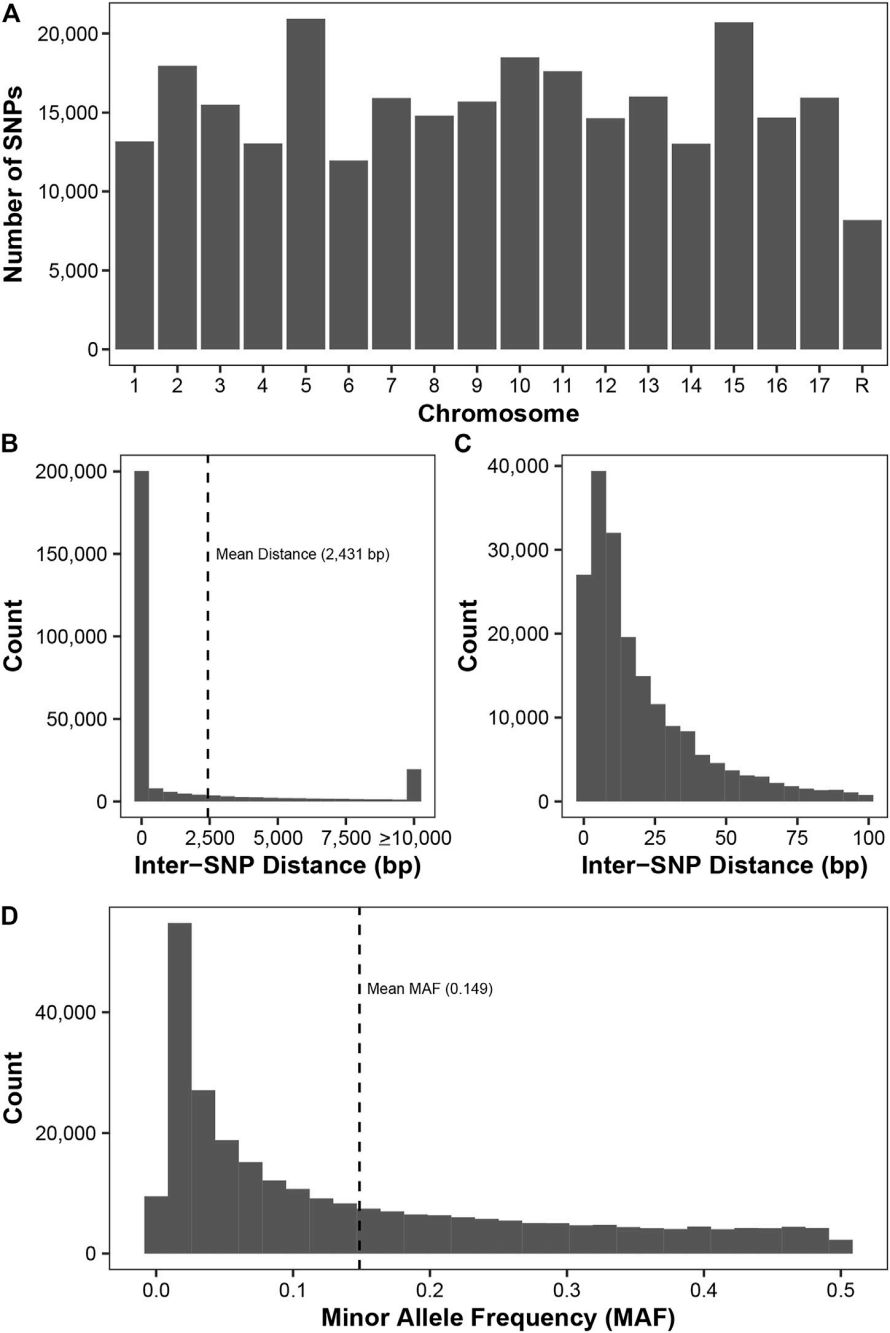
Description of the 278,231 SNPs genotyped across 11,175 apple accessions. **(A)** The number of SNPs on each chromosome, with the final chromosome (R) representing SNPs located on unassembled contigs. **(B)** The inter-SNP distance between pairs of neighbouring SNPs. SNPs on the unassembled contigs were removed prior to this analysis. The mean distance between neighbouring SNPs (2,431 bp) is indicated. **(C)** A zoom-in of plot **(B)** showing the inter-SNP distance for pairs of SNPs less than 100 bp apart. **(D)** Minor allele frequency (MAF) distribution for all SNPs. The mean MAF (0.149) is indicated.

By using three SNP callers and imputation, we were able to genotype 278,231 SNPs from 1,175 diverse apple accessions from Canada’s Apple Biodiversity Collection. These SNPs are distributed fairly evenly across all 17 apple chromosomes (Figure 1A) with the fewest number (8,187) found on unassembled contigs. There were over 11,000 SNPs identified on all chromosomes, with over 15,000 found on chromosomes 3, 9, 7, 17, 13, 11, 2, 10, 15, and 5.

The minimum inter-SNP distance between SNPs on the same chromosome was 1 bp, while the maximum distance was 1,469 kb (Figure 1B). Since sequencing was performed using 100 bp Illumina reads and SNP density is high in apple, we frequently observed more than one SNP per read. Thus, we frequently observed inter-SNP distances of less than 100 bp: over 71% of the inter-SNP distances were less than or equal to 100 bp (Figure 1C). Across the 278,231 SNPs, the average MAF was 0.149, with a high frequency of rare markers with a MAF less than 0.05 (Figure 1D).

**FIGURE 2:**
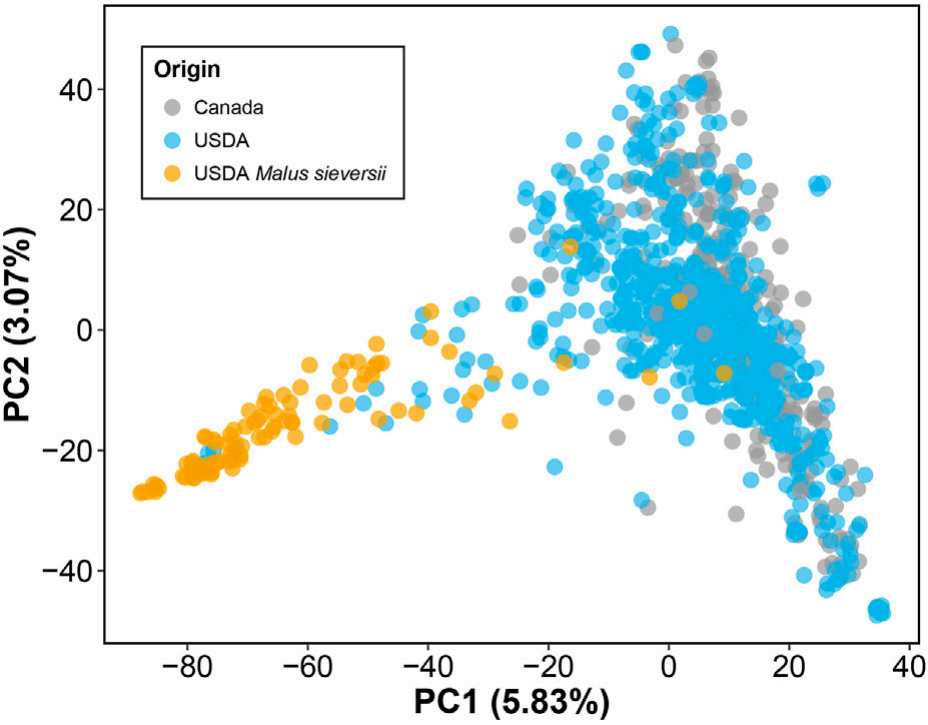
Genomic PCA of 1,175 apple accessions. PCA was performed using 180,075 LD-pruned SNPs. PC1 vs. PC2 is plotted, with the amount of variance explained by each PC indicated in parentheses. Accessions are labeled based on origin: Canada (gray) and USDA (blue). Accessions are primarily *Malus domestica,* but 98 accessions originating from the USDA are identified as *Malus sieversii* (orange).

Lastly, by performing PCA of the genotype data, we observed no clear differentiation between the *M. domestica* accessions from the USDA and those from Canada (Figure 2). However, the *Malus sieversii* accessions from the USDA collection, initially collected from Kazakhstan (Volk et al., 2013), were differentiated from *M. domestica* along PC1. Similar to previous studies however, these two species do not form clearly differentiated clusters in genetic space and thus share significant amounts of segregating polymorphism (Cornille et al., 2012; Migicovsky and Myles, 2017). It also may be the case that some *M. domestica* are incorrectly identified as *M. sieversii*, as well as the reverse.

In summary, by performing GBS for the ABC, our work provides a valuable data set for researchers working on apple genomics and improvement. By pairing these genomic data with a living germplasm collection and including approximately 100 wild apple accessions, these data are also useful for conservation-related purposes. In the future, these data can be further paired with phenotype (trait) data for genetic mapping and for identifying accessions of use in apple breeding. Lastly, by including genetic markers related to texture, fruit flavor, and disease resistance, these data may benefit those involved in genomics-assisted breeding of apples.

## Data Availability

The complete GBS SNP set of 278,231 SNPs genotyped across 1,175 apple accessions is available on Dryad: https://doi.org/10.5061/dryad.zkh1893cd. The individual imputed VCF files from each of the three SNP callers as well as the HRM data prior to imputation are also included in the Dryad upload. The raw sequence data have been deposited in the Short Read Archive under NCBI Bioproject ID PRJNA636391.

## Supplementary material

SUPPLEMENTARY FIGURE S1 Visual summary of the SNP-calling and imputation pipeline used for the data in this study. Further details are included in the Materials and Methods section.

SUPPLEMENTARY TABLE S1 Name, sequence, and target for HRM primers used to genotype 8 SNPs of interest for apple breeding in this study.

SUPPLEMENTARY TABLE S2 Metadata description of the 1,175 apple accessions genotyped for this study. The file includes the ABC apple_id (accession ID), cultivar name (PLANTID), origin of the accession (Canada, or originating from the USDA collection), ACCID/ACP/ACNO information from the USDA Germplasm Resource Information Network (GRIN) database, species information, and genomic PCs 1 to 10 based on the LD-pruned SNP set described in the study.
