# Supplementary figures and images for: Genotyping-by-sequencing of Canada’s apple biodiversity collection
Source: Front Genet. 2022 Aug 25;13:934712. doi: 10.3389/fgene.2022.934712 (PMC9452695; doi:10.3389/fgene.2022.934712)

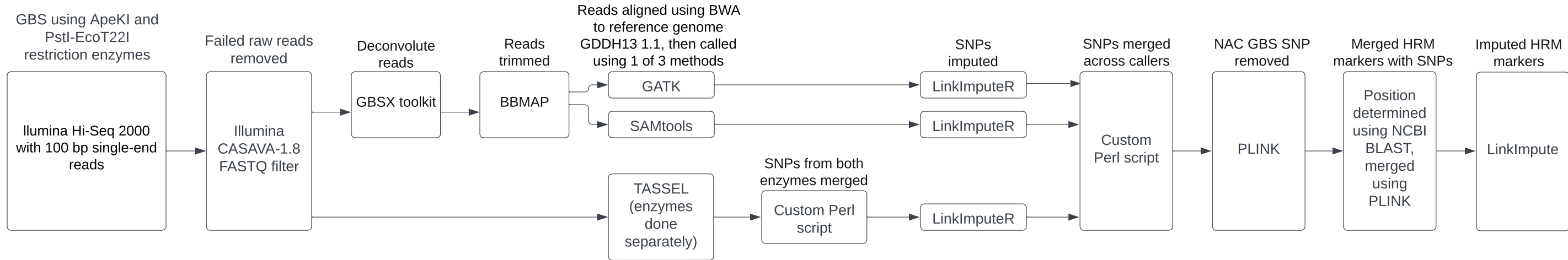

Supplement: Supplementary file 1 [file DataSheet1.zip › SUPPLEMENTARY_MATERIAL/SUPPLEMENTARY FIGURE S1.pdf]
